# Analysis of the Tunicamycin Biosynthetic Gene Cluster of Streptomyces chartreusis Reveals New Insights into Tunicamycin Production and Immunity

**DOI:** 10.1128/AAC.00130-18

**Published:** 2018-07-27

**Authors:** David Widdick, Sylvain F. Royer, Hua Wang, Natalia M. Vior, Juan Pablo Gomez-Escribano, Benjamin G. Davis, Mervyn J. Bibb

**Affiliations:** a Department of Molecular Microbiology, John Innes Centre, Norwich Research Park, Norwich, United Kingdom; b Department of Chemistry, University of Oxford, Oxford, United Kingdom

**Keywords:** tunicamycin, biosynthesis, immunity, antibiotic, Streptomyces

## Abstract

The tunicamycin biosynthetic gene cluster of Streptomyces chartreusis consists of 14 genes (*tunA* to *tunN*) with a high degree of apparent translational coupling. Transcriptional analysis revealed that all of these genes are likely to be transcribed as a single operon from two promoters, *tun*p1 and *tun*p2. In-frame deletion analysis revealed that just six of these genes (*tunABCDEH*) are essential for tunicamycin production in the heterologous host Streptomyces coelicolor, while five (*tunFGKLN*) with likely counterparts in primary metabolism are not necessary, but presumably ensure efficient production of the antibiotic at the onset of tunicamycin biosynthesis. Three genes are implicated in immunity, namely, *tunI* and *tunJ*, which encode a two-component ABC transporter presumably required for export of the antibiotic, and *tunM*, which encodes a putative *S*-adenosylmethionine (SAM)-dependent methyltransferase. Expression of *tunIJ* or *tunM* in S. coelicolor conferred resistance to exogenous tunicamycin. The results presented here provide new insights into tunicamycin biosynthesis and immunity.

## INTRODUCTION

**FIG 1:**
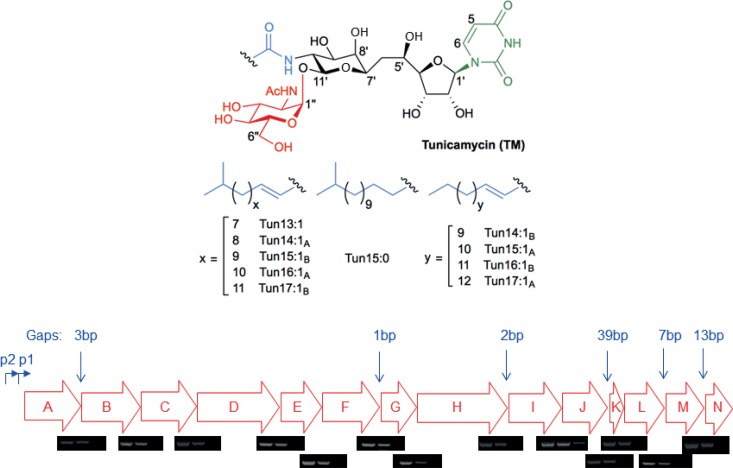
(Top) Structures of the tunicamycins. (Bottom) The tunicamycin biosynthetic gene cluster, showing the size of the intergenic regions (in bp) and the approximate location of the *tun* p1 and p2 promoters. The results of each of the reverse transcription (RT)-PCRs are shown below the corresponding intergenic region. Left lane, pIJ12003a DNA template; center lane, +RT; and right lane, −RT (control for DNA contamination).

The tunicamycins are fatty acyl nucleoside antibiotics produced by several actinomycetes, mostly Streptomyces species, including Streptomyces chartreusis (1, 2). They consist of a unique 11-carbon core (tunicamine) decorated with uracil, *N*-acetylglucosamine (GlcNAc), and variable fatty acyl moieties (Fig. 1). They are potent inhibitors of cell wall biosynthesis in Gram-positive bacteria, where they target MraY, which is required for the production of the peptidoglycan precursor Lipid I (3, 4), and TarO, MnaA, and Cap5P, which are involved in teichoic acid biosynthesis (5). They also inhibit protein *N*-glycosylation in eukaryotes (6), targeting dolichyl-phosphate alpha-*N*-acetyl-glucosaminyl-phosphotransferase (DPAGT1, also known as GlcNAc-1-P transferase) and resulting in severe toxicity. While the bacterial (e.g., MraY) and human (DPAGT1) targets are similar, in principle, it may be possible to design tunicamycin variants that specifically inhibit the bacterial proteins. A better understanding of tunicamycin biosynthesis and of the genes responsible for the individual steps in its production could play an important role in the delivery of such analogues. In an earlier work, we cloned and sequenced the tunicamycin biosynthetic gene cluster (*tun*) from S. chartreusis, expressed it heterologously in Streptomyces coelicolor, and proposed a biosynthetic pathway based largely on homology of the encoded gene products with proteins of known function (7). The cluster contains 14 genes, *tunA* to *tunN*, with many apparently translationally coupled to the preceding gene (Fig. 1). Subsequent *in vitro* studies of TunA and TunF combined with the deletion of *tunB* provided experimental evidence for their specific roles in tunicamycin biosynthesis (8). Here, we report mutational analyses of the other 13 genes in the *tun* cluster, which, together with transcriptional characterizations, provide new insights into tunicamycin biosynthesis and immunity.

## RESULTS

### Transcriptional analysis of the *tun* gene cluster.

Inspection of the *tun* gene cluster (Fig. 1) revealed that of the 14 genes, 10 appeared to be translationally coupled to the gene upstream. This, together with a maximal intergenic spacing of 39 bp, suggested that the entire *tun* gene cluster might be expressed in a single operon. To address this possibility, RNA was prepared from a lawn of S. coelicolor M1152 containing the cloned *tun* gene cluster present on a 12.9-kb SacI fragment of pIJ12003a (7) and used in reverse transcription (RT)-PCR experiments with primer pairs corresponding to *tun* sequences located approximately 100 nt from the ends and beginnings of adjacent genes (see Table S1 in the supplemental material). Amplification of cDNA spanning each of the gene junctions (Fig. 1) was indeed consistent with transcription of the *tun* genes in a single operon.

**FIG 2:**
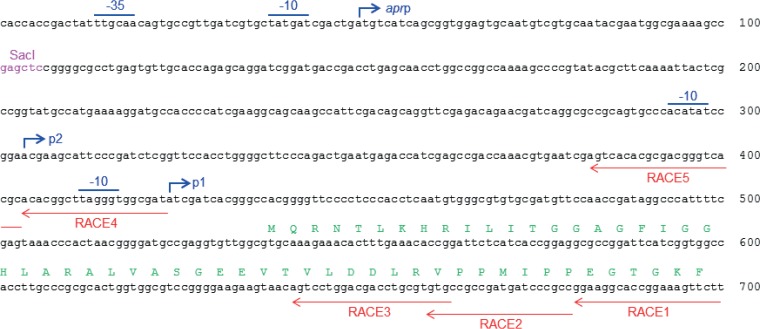
Sequence of the *tun* promoter region showing the transcriptional start sites identified by 5′ RACE. Putative −10 and −35 regions, where discernible, are overscored in blue. The locations of the complementary primers used for 5′ RACE are indicated beneath the sequence by red arrows. Nucleotides 1 to 100 are derived from the cloning vector pRT802, used to construct pIJ12003a, and the SacI site at the end of the cloned *tun* gene fragment is shown in magenta. The N-terminal amino acid sequence of TunA is shown in green. *apr*p, transcriptional start site of the apramycin resistance (*apr*) gene; p1 and p2, transcriptional start sites of the putative *tun* operon.

**FIG 3:**
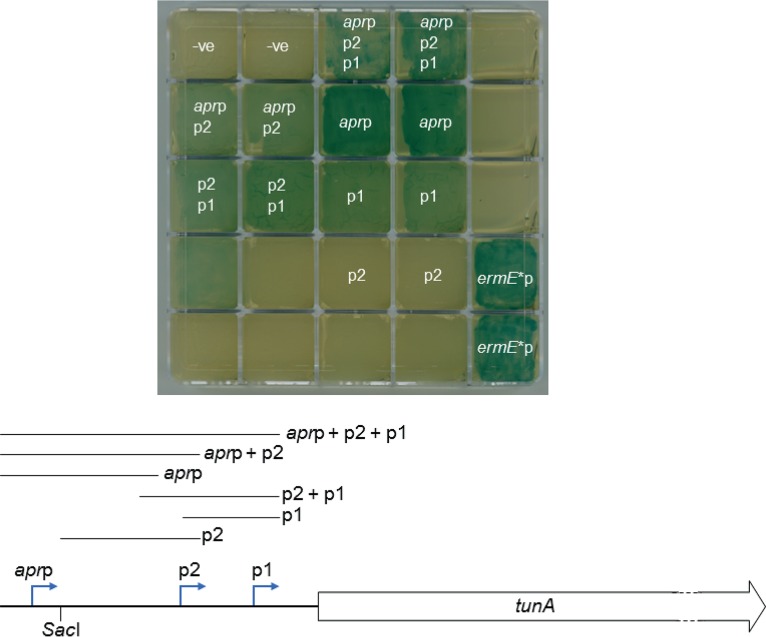
(Top) Gus reporter assays on R5 agar of promoter fragments initiating transcription of the *tun* gene cluster in S. coelicolor M1152 (negative, pGUS without an insert). Putative promoters contained within the inserted PCR fragments are indicated by *apr*p (putative promoter of the apramycin resistance gene *apr*) and p1 and p2, putative promoters of the likely *tun* operon. Gus activity results in the production of an insoluble indigo-blue precipitate, which appears green on yellow R5 agar plates. (Bottom) Schematic showing the relative positions of the three identified transcriptional start sites and the extent of the sequences present in each inserted PCR fragment. *apr*p, transcriptional start site of the apramycin resistance gene (*apr*); p1 and p2, transcriptional start sites of the putative *tun* operon.

To locate the potential transcriptional start site(s) of this likely *tun* operon, 5′ rapid amplification of cDNA ends (RACE) experiments were carried out using the S. coelicolor RNA preparation, and also RNA from S. chartreusis using five different primers, RACE1 to RACE5 (Table S1). Two putative transcriptional starts sites (*tun*p1 and *tun*p2) were identified in RNA isolated from S. chartreusis and located within the SacI fragment; while the *tunp2* transcript was not observed in RNA from S. coelicolor/pIJ12003a, transcription initiation at the promoter, *apr*p, of the apramycin resistance gene present in the cloning vector was detected (Fig. 2). To assess whether these 5′ transcript ends represented promoter activity *in vivo* (as opposed to mRNA processing or degradation), PCR fragments were generated that contained each individual putative promoter element, inserted into the β-glucuronidase reporter plasmid pGUS (9) and introduced into S. coelicolor M1152. Growth on R5 agar containing the β-glucosiduronic acid-derived substrate X-gluc confirmed promoter activity for *tun*p1 and *apr*p, but not for *tun*p2 (Fig. 3), consistent with the lack of a detectable *tun*p2 transcript in S. coelicolor M1152/pIJ12003a.

Antibiotic production in S. coelicolor under conditions of nitrogen limitation is dependent on the intracellular signaling molecular ppGpp (10). To assess whether *tun* gene transcription was dependent on ppGpp, the same β-glucuronidase promoter fusions were introduced into S. coelicolor M145 and its Δ*relA* mutant M571, and the resulting exconjugants were assayed for promoter activity on supplemented minimal medium solid (SMMS) agar containing X-gluc; no differences in promoter activity were observed between the two strains (data not shown).

### Deletion analysis of the *tun* gene cluster.

Previous work showed that deletion of *tunB* abolished tunicamycin production and provided new insights into its likely role in tunicamycin biosynthesis (8).

**FIG 4:**
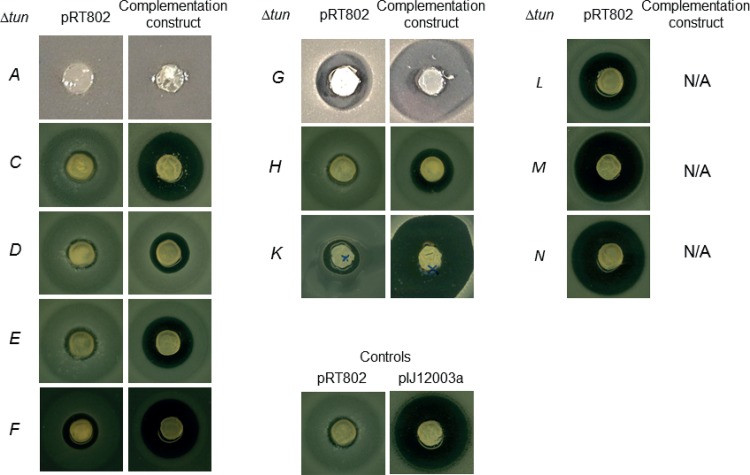
Bioassays of S. coelicolor M1152 derivatives containing a mutated *tun* gene cluster together with the empty vector pRT802 (left) or complementation construct (right). M1152 derivatives containing pIJ12003a or pRT802 as positive and negative controls, respectively, are also shown (center bottom). N/A, not applicable.

To assess the validity of the rest of the proposed tunicamycin biosynthetic pathway, in-frame deletion mutations were made by PCR-targeting for all 13 of the remaining putative biosynthetic genes, and the mutated plasmid derivatives were introduced into S. coelicolor M1152 by conjugation from Escherichia coli. The resulting strains were then assayed for antimicrobial activity, using Bacillus subtilis EC1524 as an indicator strain (Fig. 4). Individual deletion of *tunACDEH* abolished activity, while individual oblation of *tunFGK* significantly or markedly reduced it; mutations in *tunLMN* had no significant effects on the sizes of the zones of inhibition. Liquid chromatography-mass spectrometry (LC-MS) analyses of culture supernatants obtained from the individual *tunFGKLMN* mutants confirmed retention of tunicamycin biosynthesis (very low for *tunF*), while supernatants from the mutants lacking antimicrobial activity failed to show the typical characteristic tunicamycin mass spectrum of the wild-type gene cluster (see reference 7; data not shown).

To confirm that the loss of antibiotic production in the *tunACDEH* mutants and the reduction in the *tunFGK* deletion strains reflected the in-frame deletion of individual genes, and not polar effects on the expression of downstream genes, PCR fragments containing each of the deleted genes were cloned individually in pIJ12551 (*tunACDEGHK*) or in pSET152 (*tunF*) under the control of the constitutive ermE* promoter and introduced into the S. coelicolor derivative containing the corresponding mutated *tun* gene cluster. With the exception of *tunA* (see Discussion), antimicrobial activity against B. subtilis was restored in the nonproducing strains and was markedly increased in the *tunFGK* mutants (Fig. 4), confirming that the loss or reduction of antibiotic activity did indeed reflect the inactivation of individual genes.

To identify possible biosynthetic intermediates, culture supernatants and acid extracts of the mycelia of the *tunCDEH* mutants were also assessed by mass spectrometry for the production of the predicted biosynthetic intermediates that would be expected to accumulate based on the proposed biosynthetic pathway. However, none of the predicted molecular ions could be detected (data not shown). A comparative untargeted metabolomics analysis (11) of these strains also failed to show differential accumulation in any of the mutants of any metabolites with masses compatible with tunicamycin-related molecules.

An attempt was then made to identify the production of tunicamycin biosynthetic intermediates by cross-feeding and coculture experiments, using all possible pairwise combinations of mutants (including the *tunB* mutant) in which antibiotic activity had been lost. Bioactivity was assessed by overlaying the cross-feeding and coculture plates with a lawn of B. subtilis cells. In no case was antibiotic production restored (data not shown).

### Immunity to tunicamycin.

In addition to making in-frame deletion mutations in the *tun* genes thought to be involved in tunicamycin biosynthesis, we also attempted to make similar mutations in *tunIJ*, encoding a putative ABC transporter and believed to be required for export of the intracellular antibiotic. Attempts were made to make in-frame deletions of *tunIJ* together and of each gene individually, and the effect of the mutations on tunicamycin production was assessed as before, using B. subtilis EC1524 as the indicator strain. In contrast to the other *tun* gene mutations, exconjugants with deletions of *tunIJ* or *tunI* took up to 2 weeks to emerge and were few in number.

**FIG 5:**
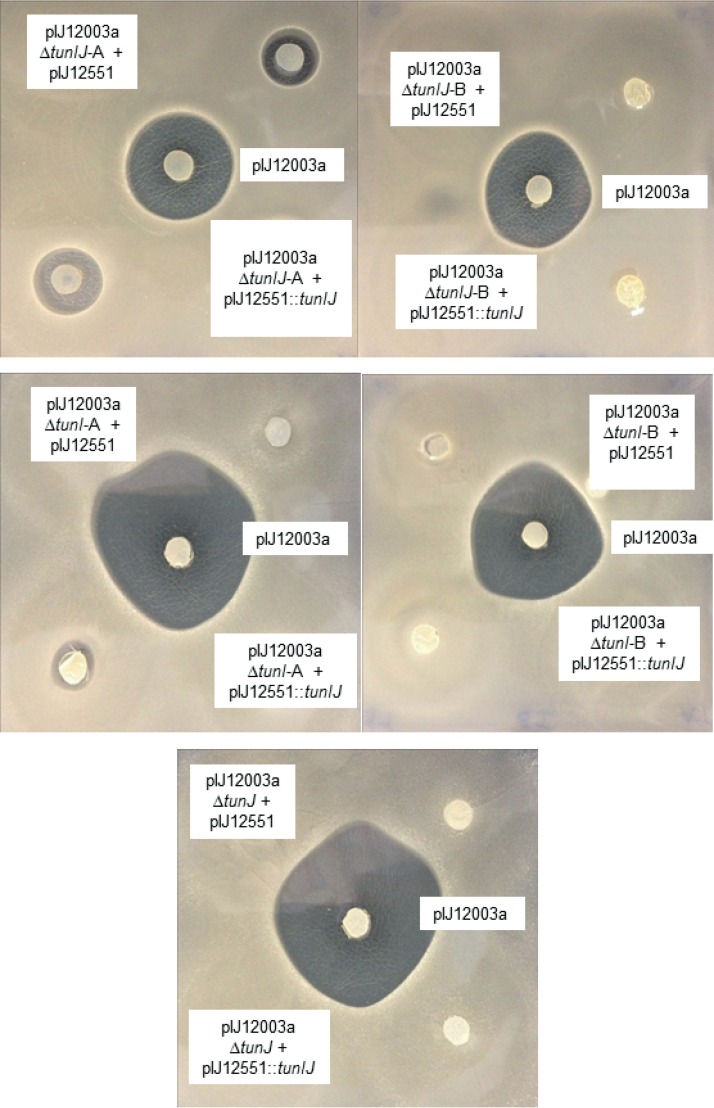
Bioassays of agar plugs of S. coelicolor M1152 containing the wild-type *tun* gene cluster (pIJ12003a), and failed attempts to complement the *tunIJ*, *tunI*, and *tunJ* mutants with wild-type versions of the deleted gene(s). In each case, B. subtilis EC1524 was used as the indicator strain. pIJ12551, the empty vector used in the complementation experiments. In panel B, note that while the pIJ12003a Δ*tunI*-A mutant clone generally gave a small zone of inhibition (see Fig. S2 in the supplemental material), it failed to do so in this particular assay.

When both genes were deleted simultaneously, two mutant phenotypes were obtained, a marked reduction in antibiotic activity (the Δ*tunIJ*-A phenotype) and a complete loss of activity (the Δ*tunIJ*-B phenotype). Introduction of pIJ12551::*tunIJ* into each of the mutants had no significant effect on their phenotypes (Fig. 5a).

Sequencing of the *tun* gene cluster in the Δ*tunIJ*-A mutant revealed insertion of a T toward the end of *tunG* (immediately after the codon for aspartate 171 of the 203-amino acid [aa] TunG) that would result in the production of a TunGH fusion protein. While earlier deletion of *tunG* reduced tunicamycin production, mutation of *tunH*, which encodes a putative UDP-tunicaminyl-uracil pyrophosphatase, abolished it. Thus, the frameshift mutation may have resulted in a fusion protein with little or no TunH activity and may have also markedly reduced or abolished translation initiation at the natural *tunH* start codon, thus resulting in markedly reduced levels of tunicamycin production.

Sequencing of the *tun* gene cluster in the Δ*tunIJ*-B mutant revealed a frameshift mutation in *tunD*, which is predicted to encode the glycosyltransferase required for addition of an *N*-acetylglucosamine moiety to tunicaminyl-uracil. The insertion of a G after the codon for threonine 279, while leaving glycine 280 and proline 281 unchanged, would replace the C-terminal 191-aa residues of the 472-aa TunD protein with 107 residues of presumably nonfunctional protein (see Fig. S1 in the supplemental material), likely accounting for the lack of bioactivity and failure to complement the mutation.

In-frame deletion of *tunI* alone yielded the same two mutant phenotypes (A and B, marked reduction in and complete loss of antibiotic activity, respectively) observed as when *tunI* and *tunJ* were deleted simultaneously; introduction of pIJ12551::*tunI* into each of the mutants had no significant effect on their phenotypes (Fig. 5b). Sequencing of the *tun* gene cluster in the *tunI*-A mutant revealed a single nucleotide change, a T to C transition found four nucleotides upstream of the GTG start codon of *tunA*. To confirm that this mutation was indeed responsible for the low level of antibiotic activity, this same mutation was introduced into pIJ12003a by replacing the 2.4-kb SacI-StuI region of the wild-type *tun* gene cluster with a PCR fragment generated from the mutated plasmid. The resulting construct (pBDW177) was confirmed by sequencing and introduced into S. coelicolor M1152, whereupon it gave the same phenotype as the *tunI*-A mutant, i.e., a much-reduced level of antimicrobial activity (see Fig. S2 in the supplemental material). Sequencing of the *tun* gene cluster in the Δ*tunI*-B mutant revealed a G to A missense mutation in *tunC* that would result in a Gly to Asp substitution at position 70 of the putative 318-aa *N*-acyltransferase, presumably resulting in loss of enzyme function and lack of tunicamycin production.

In-frame deletion of *tunJ* alone resulted in loss of antibiotic production that could not be complemented by introduction of pIJ12551::*tunJ* (Fig. 5c); subsequent sequencing of the *tun* gene cluster in two of these possibly clonal mutants revealed the insertion of a copy of IS*10* toward the end of *tunD* (one nucleotide after the codon for glycine 436 of TunD) that must have occurred when pIJ12003a was passaged through E. coli for mutant construction. Insertion of IS*10* occurred after nucleotide 12871 of GenBank accession number HQ172897 and resulted in the duplication of residues 12863 to 12871. The time taken for the emergence of the S. coelicolor M1152 exconjugants containing the *tunJ* deletion was normal for an E. coli-Streptomyces conjugation, in contrast to the prolonged period required for emergence of the *tunI* suppressor mutants, and was consistent with insertion of IS*10* into *tunD* in E. coli.

In all five cases, the unexpected mutations were presumably not only responsible for the lack of bioactivity, but also for the failure to complement the *tunIJ* mutants with wild-type copies of the genes.

### Both *tunIJ* and *tunM* confer immunity to tunicamycin in S. coelicolor.

**FIG 6:**
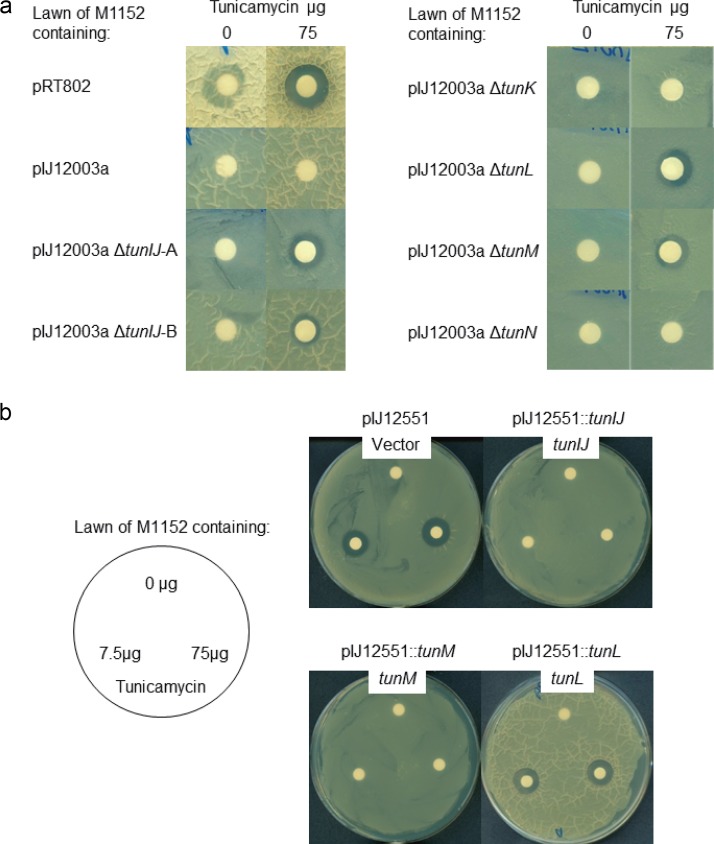
Assays of sensitivity to tunicamycin. (a) Filter paper discs containing 0 or 75 μg tunicamycin were laid on top of lawns of spores of S. coelicolor M1152 derivatives containing the wild-type *tun* gene cluster (pIJ12003a) and various deleted versions. Deletion of *tunIJ*, *tunL*, or *tunM* resulted in increased sensitivity to tunicamycin. (b) Filter paper discs containing 0, 7.5, or 75 μg tunicamycin were laid on top of lawns of spores of S. coelicolor M1152 derivatives containing the ermE*p expression vector pIJ12551 or derivatives thereof containing *tunIJ*, *tunM*, or *tunL*. In both sets of assays, R5 agar was used, and the plates were incubated at 30°C for 48 h.

The results obtained above suggested that in addition to playing a role in the export of tunicamycin, *tunIJ* also played a role in conferring immunity to the antibiotic in the producing organism, and that their deletion resulted in lethality or the selection of mutations that abolished or markedly reduced the level of tunicamycin production. To assess the potential role of these genes in immunity, S. coelicolor derivatives containing the wild-type *tun* gene cluster and derivatives from which *tunIJ*, *tunK*, *tunL*, *tunM*, or *tunN* had been deleted were plated as lawns on R5 agar, and their susceptibility to tunicamycin was assessed. While the wild-type gene cluster conferred complete immunity to exogenous tunicamycin, deletion of *tunIJ* and, surprisingly, of *tunL* and *tunM*, resulted in increased sensitivity, although all three deletion mutants were noticeably more resistant than the strain containing the empty vector (pRT802) (Fig. 6a). Deletion of *tunK* or *tunN* had no effect on susceptibility to tunicamycin.

To assess whether expression of *tunIJ*, *tunL*, or *tunM* alone could confer immunity to tunicamycin in S. coelicolor, the pIJ12551 expression constructs containing each of the genes that had been used in the earlier complementation assays were introduced into S. coelicolor M1152 by conjugation, and the resulting strains were used in tunicamycin susceptibility assays. Expression of either *tunIJ* or *tunM* from the ermE* promoter conferred complete immunity, while expression of *tunL* resulted in the same level of susceptibility as that of the vector control (Fig. 6b). We therefore assume that the enhanced susceptibility to tunicamycin shown by the *tunL* deletion mutant observed above reflected a polar effect on *tunM* expression.

## DISCUSSION

Although the presence of promoter elements within the cluster cannot be excluded, the results presented in this paper suggest that all of the genes in the tunicamycin biosynthetic gene cluster are cotranscribed in S. chartreusis from two promoters, one of which, *tun*p2, is not utilized in S. coelicolor. The latter presumably reflects elements of regulation that are missing in the heterologous host or the absence of *cis*-acting regulatory sequences upstream of *tun*p2 in the cloned SacI fragment that are required for its activation. With the exception of the 5′ *tunA*, all of the genes required for wild-type levels of tunicamycin biosynthesis appear to be translationally coupled to the preceding gene. It is conceivable that such an arrangement ensures near stoichiometric production of each of the corresponding proteins, which, interestingly, would be consistent with the production of a large multifunctional enzyme complex. Given that *tunIJ* also exhibit this coupling, it is also conceivable that such a complex could be located at the cell membrane. Its existence would also be consistent with the inability to detect biosynthetic intermediates predicted to accumulate in the *tunCDEH* mutants either by mass spectrometry analysis or by cross-feeding, i.e., the lack of any one component could result in inactivation of the entire complex (or indeed of a subcomplex).

Repeated attempts to complement the *tunA* mutant using both the ermE* and native *tunA* promoter (data not shown) failed. Similar observations were made for *mibA*, the 5′ gene in a polycistronic mRNA that is essential for microbisporicin biosynthesis in Microbispora corallina (Lucy Foulston, personal communication). It is conceivable that this reflects an essential element of posttranscriptional control that operates on the 5′ end of the *tun* operon transcript that is absent in the *tunA* deletion mutant.

**FIG 7:**
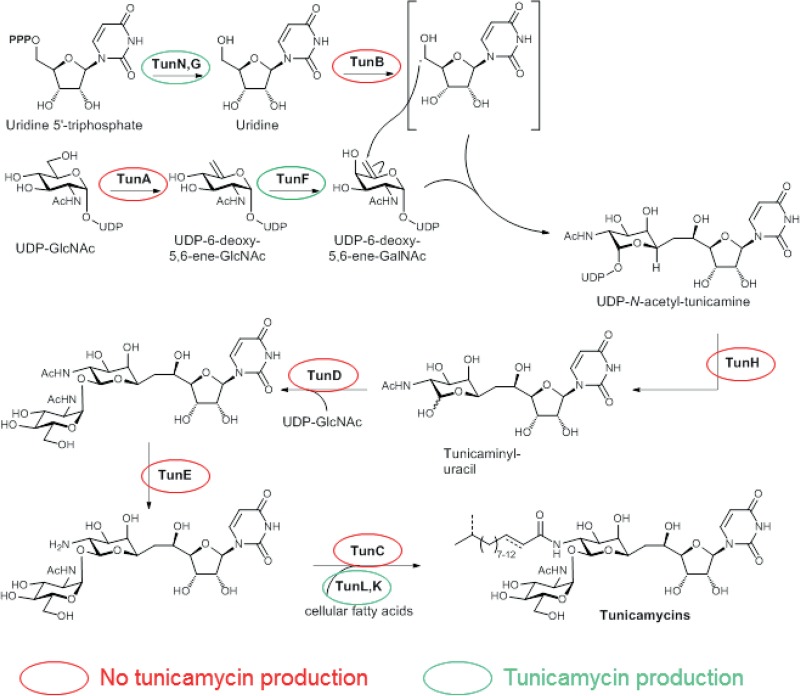
Proposed pathway for tunicamycin biosynthesis. *tun* gene products essential for tunicamycin production are circled in red and nonessential proteins in green.

The deletion analysis reported here and previously (8) has demonstrated that of the 14 genes contained within the *tun* gene cluster just six (*tunABCDEH*) are essential for tunicamycin production in S. coelicolor, each of which can be assigned a likely biosynthetic role Fig. 7) (7, 8), while *tunI* and *tunJ* are required for immunity. Of the others, *tunFGKLN* all have putative roles also carried out by homologues involved in primary metabolism, potentially explaining their nonessentiality. This would require that the primary metabolic enzymes were capable of substituting for their Tun counterparts if the latter were indeed present in the hypothetical tunicamycin biosynthetic complex (see above); alternatively, the complex might only contain the essential enzymes, TunABCDEH. The presence of *tunFGKLN* within the *tun* gene cluster may be to ensure adequate precursor supply and/or flux at the onset of tunicamycin production, feeding the likely critical C-C bond-forming step (*tunFGN*) and efficient acylation from the endogenous fatty acid pool during biosynthesis (*tunKL*) (see Fig. 7 and references 7, 8 for a detailed discussion of tunicamycin biosynthesis). Indeed, deletion of *tunF*, *tunG*, or *tunK* resulted in a reproducible reduction in antibiotic activity (Fig. 4). The ability of the *tunL* mutant clone to produce tunicamycin in S. coelicolor M1152 contrasts with the results reported by Chen et al. (12), where tunicamycin production was abolished when a *tunL*-deleted gene cluster was introduced into Streptomyces lividans TK24. However, since TunL is a putative phospholipid phosphatase with homologues encoded by the S. coelicolor genome (e.g., SCO1102 and SCO0402) that are likely involved in primary metabolism, it is conceivable that this disparity reflects differences in the heterologous hosts and growth conditions used in the two studies. Given the very low level of tunicamycin production in the *tunF* mutant, it is also possible that a homologous host epimerase (e.g., SCO3137 and/or SCO2988) is responsible for partial suppression of *tunF* oblation. The deletion analysis reported here rules out the previously assigned role for TunM in tunicamycin biosynthesis (8). However, the ability of *tunM* to confer resistance to tunicamycin in S. coelicolor, and the increased levels of tunicamycin susceptibility observed after deletion of *tunM* from the *tun* gene cluster in S. coelicolor, suggest that it may instead play a role in immunity in the producing organism. Intriguingly, TunM encodes a putative SAM-dependent methyltransferase and, although unprecedented to our knowledge, it is conceivable that it mediates its effect by modification of intracellular tunicamycin to provide an immunity mechanism, although such a nonreversible modification would require a corresponding demethylation for activation, perhaps after export. Conceivably, such demethylation enzymes might exist in the natural host's key target organisms. Alternatively, self-resistance might be mediated by methylation of endogenous enzyme (or other molecular) targets of the antibiotic (or indeed by modification of one of their substrates in a manner that would afford sufficient substrate protection to prevent tunicamycin from binding to any corresponding complex).

## MATERIALS AND METHODS

### Strains and general methods.

The strains used in this study are listed in Table 1. *Escherichia coli* strains were grown and manipulated following standard methods (14, 23), with *E. coli* DH5α (15) used as the general cloning host. Bacillus subtilis EC1524 (13) was grown in Luria Bertani broth (23). Streptomyces strains were grown and manipulated as described previously (7, 24). Plasmids and oligonucleotides are described in Tables 1 and S1, respectively. Tunicamycin was obtained from Abcam Biochemicals and dissolved in 100% dimethyl sulfoxide (DMSO) at a concentration of 5 mg/ml prior to use.

**TABLE 1:** Strains and plasmids used and/or created in this study

Strain or plasmid	Description	Reference and/or source
Strains		
B. subtilis EC1524	Bioassay strain	13
E. coli BW25113/pIJ790	E. coli containing λ Red plasmid	14
E. coli DH5α	General cloning host	15
E. coli BT340	FLP recombinase strain	14
E. coli ET12567/pUZ8002	Conjugation of plasmids into S. coelicolor M1152	16
E. coli ET12567/pR9406	Conjugation of plasmids into S. coelicolor M1152	17; David Figurski, personal communication
S. coelicolor M1152	Heterologous expression strain	18
Plasmids		
pBlueScript II KS	General cloning vector	Agilent Technologies
pGUS	β-glucuronidase reporter plasmid	9
pIJ773	PCR template for apramycin resistance cassette	14
pIJ10257	ϕBT1 integrative vector; used as source of ermE*p	19
pIJ12003a	pRT802 containing the *tun* cluster on a 12.9 kb SacI fragment	7
pIJ12541	pIJ12003a with *tunB* deleted	8
pIJ12551	ϕC31 integrative expression vector with ermE* promoter	20
pRT802	ϕBT1 integrative vector	21
pSET152	ϕC31 integrative vector	22
pBDW91	Effectively pIJ12003a with *tunA* deleted	This study
pBDW92	Effectively pIJ12003a with *tunC* deleted	This study
pBDW36	Effectively pIJ12003a with *tunD* deleted	This study
pBDW37	Effectively pIJ12003a with *tunE* deleted	This study
pIJ12542	pIJ12003a with *tunF* deleted	This study
pBDW38	Effectively pIJ12003a with *tunG* deleted	This study
pBDW39	Effectively pIJ12003a with *tunH* deleted	This study
pBDW40	Effectively pIJ12003a with *tunI* deleted	This study
pBDW41	Effectively pIJ12003a with *tunJ* deleted	This study
pBDW42	Effectively pIJ12003a with *tunIJ* deleted	This study
pBDW43	Effectively pIJ12003a with *tunK* deleted	This study
pBDW44	Effectively pIJ12003a with *tunL* deleted	This study
pBDW45	Effectively pIJ12003a with *tunM* deleted	This study
pBDW46	Effectively pIJ12003a with *tunN* deleted	This study
pBDW58	pIJ12551 *tunA* complementation construct	This study
pBDW59	pIJ12551 *tunC* complementation construct	This study
pBDW60	pIJ12551 *tunD* complementation construct	This study
pBDW61	pIJ12551 *tunE* complementation construct	This study
pIJ12544	pSET152 ermE*p::*tunF* complementation construct	This study
pBDW62	pIJ12551 *tunG* complementation construct	This study
pBDW65	pIJ12551 *tunH* complementation construct	This study
pBDW66	pIJ12551 *tunIJ* complementation construct	This study
pBDW155	pIJ12551 *tunK* complementation construct	This study
pBDW132	pIJ12551 *tunL* complementation construct	This study
pBDW133	pIJ12551 *tunM* complementation construct	This study
pBDW177	pIJ12003a carrying the *tunI*-A mutation in *tunA*	This study

### Transcriptional analysis.

RNA was prepared from lawns of mycelium approximately 30 mm in diameter of S. coelicolor M1152/pIJ12003a and S. chartreusis cells grown for 2 days on R5 and DNA agar (18, 24), respectively, using a bead-beater and a Qiagen RNeasy kit (Qiagen, Crawley, United Kingdom). RT-PCR analysis was carried out on the S. coelicolor RNA sample; cDNA was prepared using a Qiagen reverse transcription kit (Qiagen, Crawley, United Kingdom) and subjected to PCR, using the primer pairs listed in Table S1. pIJ12003a was used as a positive control, and RNA that had not been treated with reverse transcriptase was used as a negative control. The 5′ ends of the *tun* transcripts present in both RNA samples were identified by using a 5′ rapid amplification of cDNA ends (RACE) kit (version 2.0; Invitrogen, Paisley, United Kingdom) following the manufacturer's instructions. Briefly, first-strand cDNA synthesis was carried out using 5 μg of RNA, reverse transcriptase and the oligonucleotide primer RACE1 (Table S1). cDNA was purified using the SNAP columns provided in the kit, and poly(dC) tails were added to the 3′ ends using terminal deoxynucleotidyl transferase. PCR amplification of the tailed cDNA was initially carried out using the 5 ′ RACE abridged anchor primer with the first-strand primer RACE2 or RACE4 (Table S1). A dilution of the PCR mixture was then subjected to a second amplification using the abridged anchor primer with the second nested primer RACE3 or RACE5 (Table S1). The PCR product was gel-purified and a portion sequenced directly using the oligonucleotide RACE3 or RACE5 as primer.

### Gus assays.

The DNA fragments to be assessed for promoter activity were cloned individually as XbaI-KpnI PCR fragments in pGUS (9) and cleaved with the same two restriction enzymes, and the resulting constructs were introduced into S. coelicolor M1152, M145, and M571 (Δ*relA* mutant of M145; see reference 25) by conjugation, whereupon they integrated at the chromosomal φC31 *attB* sites of each strain. The ability of the cloned fragments to direct transcription of the *uidA* gene encoding β-glucuronidase (Gus) was determined by plating the exconjugants on R5 and SMMS agar medium (24) containing 0.16 mg/ml X-gluc (5-bromo-4-chloro-1*H*-indol-3-yl beta-d-glucopyranosiduronic acid) (cyclohexylammonium salt; Gold Biotechnology).

### Construction of deletion mutants.

To construct the *tunACDEGHIJKLMN* mutants, the 12.9-kb SacI fragment of pIJ12003a, a derivative of pRT802 (21), was first subcloned into pBlueScript II KS to give pBDW7. Gene deletions were made using the method of Gust et al. (2003) (14) by targeting pBDW7 with apramycin resistance gene (*apr*) replacement cassettes generated using the PCR primers listed in Table S1 and pIJ773 as the template DNA, and the cassettes were subsequently deleted using FLP recombinase to give in-frame deletion mutants; in all cases, the ribosome-binding site of the downstream gene was retained in the mutant construct. The SacI fragments from the resulting plasmids were subcloned into pRT802, and those with the fragment inserted in the same orientation as in pIJ12003a (determined by restriction enzyme digestion) were selected for further study. To construct the *tunF* mutant, pIJ12003a was targeted in the same manner (see Table S1 for the primers used) to yield the mutant derivative pIJ12542. Each of the mutated plasmids was introduced into E. coli ET12567/pR906 (17; David Figurski, personal communication) by transformation and then into S. coelicolor M1152 by conjugation.

### Complementation of deletion mutants.

A PCR fragment was generated for selected deleted *tun* genes using the primers in Table S1, pIJ12003a DNA as the template, and Phusion High Fidelity DNA polymerase (New England BioLabs Inc.). The PCR products for *tunACDEGHIJK* were cleaved with NdeI and PacI and inserted into pIJ12251 (20) cut with the same restriction enzymes. The PCR product for *tunF* was cleaved with NdeI and HindIII and inserted downstream of the ermE* promoter of pIJ10257 (19) that had been cut with the same restriction enzymes. The resulting plasmid was digested with BamHI and EcoRI and the ermE*p::*tunF* fragment ligated into pSET152 (22) that had been similarly treated to give pIJ12544. All of the individual complementation constructs, which were confirmed by DNA sequencing, were introduced into E. coli ET12567/pUZ8002 (16) by transformation and then into the appropriate S. coelicolor deletion strain by conjugation.

### Bioassays for tunicamycin production.

Lawns of the strains to be assayed were made by spreading approximately 10^7^ spores in 100 μl of water on R5 agar plates, followed by incubation at 30°C for 48 h. Soft nutrient agar (SNA) was melted, cooled to 55°C, and inoculated with a one-tenth volume of a mid-logarithmic growth culture of B. subtilis EC1524. Cylindrical plugs (approximately 8 mm in diameter) were cut from the Streptomyces lawns using a cork borer and were either set into or laid on top of 40 ml of the SNA inoculated *with B. subtilis* EC1524 in a 10-cm^2^ plastic petri dish, which was then incubated overnight at 30°C.

### Analysis of immunity to tunicamycin.

Lawns of the strains to be assayed were made by spreading approximately 10^7^ spores in 100 μl of water onto R5 plates, which were allowed to dry for 20 min. Filter paper discs with various amounts of tunicamycin (dissolved in DMSO) were laid onto the lawns, and the plates were incubated at 30°C for 48 h.

## Supplementary Material

Supplemental file 1
