# Supplementary material for: Analysis of the Tunicamycin Biosynthetic Gene Cluster of Streptomyces chartreusis Reveals New Insights into Tunicamycin Production and Immunity
Source: Antimicrob Agents Chemother. 2018 Jul 27;62(8):e00130-18. doi: 10.1128/AAC.00130-18 (PMC6105854; doi:10.1128/AAC.00130-18)
Supplement: Supplemental file 1 [file zac008187336s1.pdf]

Supplementary Material

Analysis of the tunicamycin biosynthetic gene cluster of *Streptomyces chartreusis* reveals new insights into tunicamycin production and immunity

David Widdick[a], Sylvain F. Royer[b], Hua Wang[b*], Natalia M. Vior,[a] Juan Pablo Gomez- Escribano[a], Benjamin G. Davis[b], Mervyn J. Bibb[a]

[a]Department of Molecular Microbiology, John Innes Centre, Norwich Research Park, Norwich, United Kingdom

[b]Department of Chemistry, University of Oxford, Oxford, United Kingdom[b]

Running title: Tunicamycin biosynthesis and immunity

Address correspondence to Mervyn J. Bibb, mervyn.bibb@jic.ac.uk.

* Present address: Hua Wang, The Francis Crick Institute, London, United Kingdom

Table S1. Oligonucleotide primers (5' to 3')

In all cases, nucleotides in lower case correspond to *tun* sequences, those in upper case to the apramycin resistance cassette (Sequences of deletion primers) or to introduced restriction sites (elsewhere).

Gene	Primer name	Sequences of deletion primers
tunA	tunAdelF<br>tunAdelR<br>tunAdelR2	gagtaaacccactaacggggatgccgaggtgttggcgtgATTCCGGGGATCCGTCGACC<br>ccagactggcgcgtacaggtgcggtgtagccggtcatcaTGTAGGCTGGAGCTGCTTC<br>caggtgcggtgtagccggtcatcagtcggcccccctcctcTGTAGGCTGGAGCTGCTTC
tunC	tunCdelF<br>tunCdelR<br>tunCdelR2	cggcagatcttcgaaggcgcggaggtgaggtaggccttgATTCCGGGGATCCGTCGACC<br>cccatacctgatcggagacagtgaagatgatctccattaTGTAGGCTGGAGCTGCTTC<br>cagtgaagatgatctccattagttccgaacacccctcccTGTAGGCTGGAGCTGCTTC
tunD	tunDdelF<br>tunDdelR	gtgcccgggaatcttcgggaggggtgttcggaactaatgATTCCGGGGATCCGTCGACC<br>cgtcatccgggtgtgcggcgatgaccaggaccttcacgcTGTAGGCTGGAGCTGCTTC
tunE	tunEdelF<br>tunEdelR	cgcggacggcgaacgaccggttacggagcccgcgtgaagATTCCGGGGATCCGTCGACC<br>ccacatacccggcacccccagtcacgagcactctcatcgTGTAGGCTGGAGCTGCTTC
tunF	tunF20<br>tunF19	ggttcccagtggagttgatgtgcgatgagagtgctcgtgATTCCGGGGATCCGTCGACC<br>gcgtggcgcagtaggtagaggtgcatgtcactccagcgaTGTAGGCTGGAGCTGCTTC
tunG	tunGdelF<br>tunGdelR	actggcacaccagtacccccgcgtcgctggagtgacatgATTCCGGGGATCCGTCGACC<br>cacagatgacgaccatacgcctgcgccgcccgctcatgcTGTAGGCTGGAGCTGCTTC
tunH	tunHdelF<br>tunHdelR	tcacggatccctgagcacagcggagttgagcgcatgagcATTCCGGGGATCCGTCGACC<br>cgacctcatatgcgtaaggagggttttctggcatgctcaTGTAGGCTGGAGCTGCTTC
tunI	tunIdelF<br>tunIdelR	cagcatctggcgccaagcggcgcgggagatgtgagcatgATTCCGGGGATCCGTCGACC<br>aagctcagtccgagcttcttgtcacgcgtagctgacataTGTAGGCTGGAGCTGCTTC
tunJ	tunJdelF<br>tunJdelR	ccgcgaccctggaagacgtgtatgtcagctacgcgtgacATTCCGGGGATCCGTCGACC<br>tgtgtttcccttcgtgatgagtgatcgatggttgcgtcaTGTAGGCTGGAGCTGCTTC
tunK	tunKdelF<br>tunKdelR	aaccatcgatcactcatcacgaagggaaacacatccatgATTCCGGGGATCCGTCGACC<br>gtgaccggggccggtcgccggagtggtttcggatcatgcTGTAGGCTGGAGCTGCTTC
tunL	tunLdelF<br>tunLdelR	gctggaccacatttcgaagtccaccagccccgcatgatcATTCCGGGGATCCGTCGACC<br>tggtaatgatcgttgtggttgaacggcacggcgtcatcaTGTAGGCTGGAGCTGCTTC
tunM	tunMdelF<br>tunMdelR	cgaactccctggacgagcccgtgtcatgatgacgccgtgATTCCGGGGATCCGTCGACC<br>cttctcggtggctgagagatcatgtgtgtccggctgctaTGTAGGCTGGAGCTGCTTC
tunN	tunNdelF<br>tunNdelR	aggcagggctgggaagtgattagcagccggacacacatgATTCCGGGGATCCGTCGACC<br>attctcgatatcgcagcccggcggcgcgcggttccttcaTGTAGGCTGGAGCTGCTTC

Gene	Primer name	Sequences of complementation primers
tunA	tunAFNde<br>tunARPac	GCCGAGATCTCATAtgcaaagaaacactttgaaacaccg<br>GCCGAAGCTTAATTAAtcagtcggccccctcctcttggaag
Native promoter	tunA+PF<br>tunA+PR	GGCGCGTCTAGAgagccgaccaaacgtgaatc<br>GGCGCGGAATTCggcgcgtacaggtgcggtgtag
tunC	tunCFNde<br>tunCRPac	GCCGAGATCTCATAtgtccagggaggccctcatacgcc<br>GCCGAAGCTTAATTAAttagttccgaacacccctcccgaaga
tunD	tunDFNde<br>tunDRPac	GCCGAGATCTCATAtggagatcatcttcactgtctccga<br>GCCGAAGCTTAATTAAtcacgcgggctccgtaaccggtcgtt
tunE	tunEFNde<br>tunERPac	GCCGAGATCTCATAtgaaggtcctggtcatcgccgcacac<br>GCCGAAGCTTAATTAAtcatcgcacatcaactccactgggaa
tunF	tunF1<br>tunF2	AACATatgagagtgctcgtgactg<br>TTAAGCTTgtagaggtgcatgtcactc
tunG	tunGFNde<br>tunGRPac	GCCGAGATCTCATAtgcacctctacctactgcgccacgc<br>GCCGAAGCTTAATTAATcatgcgctcaactccgctgtgctca
tunH	tunHRNde<br>tunHRPac	GCCGAGATCTCATAtgagcgggcggcgcaggcgtatggt<br>GCCGAAGCTTAATTAAcacatctcccgcgccgcttggcgcca
tunIJ	tunIJFNde<br>tunIJRpac	GCGGGATCCATatgccagaaaaccctccttacgcttatgaggtcgagggc<br>GCCGAAGCTTAATTAAtcacgaggcgcggcgcccgtagcggg
tunK	tunKndeF<br>tunKpacR	GGCCGGATCCATatgaccgagcaacagttcc<br>GGCCAAGCTTAATTAAtcatgcggggctggtggac
tunL	tunLndeF<br>tunLpacR	CGCGGGTACCATatgatccgaaaccactccggcgac<br>CGCGAAGCTTAATTAAtcatgacacgggctcgtccagggag
tunM	tunMndeF<br>tunMpacR	CGCGGGTACCATAtgccgttcaaccacaacgatcattac<br>CGCGAAGCTTAATTAActaatcacttcccagccctgccttc

Promoters	Primer name	Sequences of primers for pGUS derivatives
*apr*p, p2, p1	Papra-F1<br>Papra-R1	GGCGCCTCTAGAcaccaccgactatttgcaacag<br>GGCGCCGGTACCttggaacatcgcacacgcccac
*apr*p, p2	Papra-F1<br>Papra-R2	GGCGCCTCTAGAcaccaccgactatttgcaacag<br>GGCGCCGGTACCggtggaaccgagatcgggaatg
*apr*p	Papra-F1<br>Papra-R3	GGCGCCTCTAGAcaccaccgactatttgcaacag<br>GGCGCCGGTACCcctgctgtcgaatggcttgctg
p2, p1	Papra-F2<br>Papra-R1	GGCGCCTCTAGAcagcaagccattcgacagcagg<br>GGCGCCGGTACCttggaacatcgcacacgcccac
p1	Papra-F3<br>Papra-R1	GGCGCCTCTAGAcattcccgatctcggttccacc<br>GGCGCCGGTACCttggaacatcgcacacgcccac
p2	tunP2F<br>tunP2R	GGCGCGTCTAGAgagctccggggcgcctgagtg<br>GGCGCGGGTACCcttcgttccggatatgtgg

Primer name	5’ RACE primers
RACE1	agaactttccggtgccttcc
RACE2	ggcgggatcatcggcggcac
RACE3	cacacgcaggtcgtccaggact
RACE4	atcgccaccctaagccgtgt
RACE5	gcgtgacccgtcgcgtgtgact

Intergenic region	Primer name	Sequences of primers used for RT-PCR
tunAB	tunBtestF<br>tunAtestR2	ccttgcagacgcggcagatc<br>aggcgtacgtcagagagatcc
tunBC	tunCtestF<br>tunBtestR	gtctgcgcgagatcttctgg<br>ggaacgacaggagcgatatc
tunCD	tunDtestF<br>tunCtestR	gctgggaggcgtaggcaacg<br>gaagcatggctccaccctcc
tunDE	tunEtestF<br>tunDtestR	gagacgctcggtgtgattgc<br>gaatgccgcagcgagatacc
tunEF	tunFtestF<br>tunEtestR	ggcggccgttcggacgctgg<br>tatcgaggatgtccaccacg
tunFG	tunGtestF<br>tunFtestR	ctgggccgatccgagtctgg<br>cgctcagccagccaatgagc
tunGH	tunHtestF<br>tunGtestR	acacactggcagtaccgtcc<br>ggcagtgccgggaagtgtcg
tunHI	tunItestF<br>tunHtestR	caccgaattccagtacagc<br>ccattgctgcccagaagtcc
tunIJ	tunJtestF<br>tunItestR	ccaccgcggcaaggtcgtgg<br>tgctgaccaaaggggcaagc
tunJK	tunKtestF<br>tunJtestR	gcgatccttgccatctttgc<br>cagttgttcctgggagaagg
tunKL	tunLtestF<br>tunKtestR	cctcgcacccctgatcgagg<br>tctcgctcagccgttcgtcc
tunLM	tunMtestF<br>tunLtestR	gctcgctgggcaactcttgg<br>cgaagcgctagtttctgtgc
tunMN	tunNtestF<br>tunMtestR	gacagtgctgaatatcttcc<br>cgcgcaataaggacacgtcc

Wild type TunD sequence (472 aa)

```
  1 meiiftvsdq vwggkhrymh dmalglaqag htvtvlaeeg gamlqqcraa gvttvsvpaf
 61 asddaaeavr kalrhrrphi vcvsgradaa avhhaqsqgv tdaavclfrh safplgttde
121 vrdlftgvnl vfttsleqrq rqfeplinag vlkdeqveil tsgvgeplla aldaadrgaa
181 rkelraesdq fvflvlarla wekgidqvid afadlelppd aappllvvag egpleaelrg
241 qtiergvaer vqflghqdhv apvikasdav vltstvpetg plalkeamaa grpviasvqg
301 gipefveder hgllvidded lrqamqrlls dreaaetmga agsesvrggh ravrrveyla
361 hrldllaleq lapdtvlhev vwddvrlree tqggfvfvpr tshimeldsa tyavvrtave
421 agdplqlikl peetlgviah rlyamgalvr qdgqatpaar tadgerpvte pa*
```

Predicted TunD sequence (388 aa) in the *tunIJ*:B mutant

```
  1 meiiftvsdq vwggkhrymh dmalglaqag htvtvlaeeg gamlqqcraa gvttvsvpaf
 61 asddaaeavr kalrhrrphi vcvsgradaa avhhaqsqgv tdaavclfrh safplgttde
121 vrdlftgvnl vfttsleqrq rqfeplinag vlkdeqveil tsgvgeplla aldaadrgaa
181 rkelraesdq fvflvlarla wekgidqvid afadlelppd aappllvvag egpleaelrg
241 qtiergvaer vqflghqdhv apvikasdav vltstvpetg paraqgghgr rpprhrlcpg
301 rhpgvrrgra arsaghrrrg paagdaaape rprsrrdhgs rrirvgsrrt scsttrgvpr
361 ppsrspgpgt arpghgpsrs gvgrrtaa*
```

Fig. S1. Amino acid sequence of TunD and the predicted amino acid sequence of the fusion protein in the *tunIJ*:B mutant. The sequence in blue is predicted to be replaced by the sequence in red in the mutant.

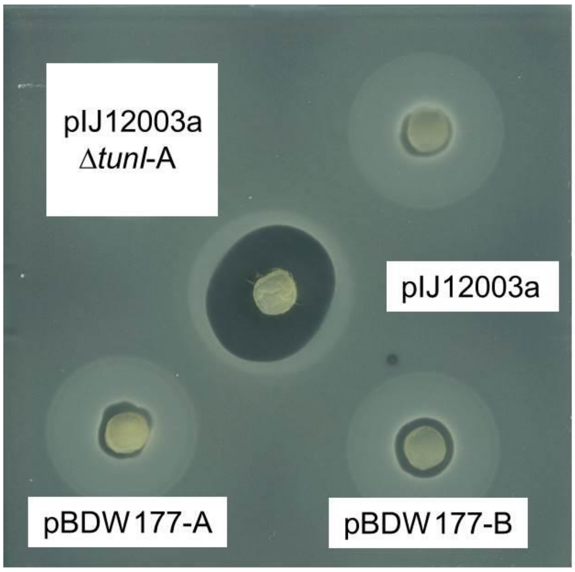

Fig. S2. Bioassays of agar plugs of *S. coelicolor* M1152 containing the wild type *tun* gene cluster (pIJ12003a), the mutant cluster obtained after deletion of *tunI* (the A phenotype; pIJ12003a Δ*tunI*-A), and the wild type cluster after introduction of the mutation identified in the pIJ12003a Δ*tunI*-A gene cluster (pBDW177-A and pBDW177-B – two independent clones). *B. subtilis* EC1524 was used as indicator strain.
